# Book Reviews

**Published:** 1799-04

**Authors:** 


### CRITICAL RETROSPECT
### OF
### *MEDICAL AND PHYSICAL LITERATURE.*

### [FOREIGN AND DOMESTIC.]

### NATURAL HISTORY AND BOTANY.

*Tableau elémentaire d'hiſtoire naturelle des animaux :* i. e. " *An elementary view of the Natural Hiſtory of Animals ;*" by Citizen CUVIER. One Volume 8vo. with fourteen plates. *Paris.* Baudouin.

In this popular and inſtructive work, the author treats ſucceſſively of the phyſical hiſtory of man, the mammalia, birds, reptiles, fiſhes, inſects and zoophytes. He explains and compares the varieties and modifications of the principal organs of animals, in as clear and ſatisfactory a manner, as our preſent knowledge of Phyſiology and Natural Hiſtory will admit. The different colour and temperature of animal blood forms an additional and highly intereſting object of Cit. Cupier's able inquiries : hence he claſſes them under the various heads of animals with red blood and white blood ; and again divides them into thoſe having warm and red blood, and thoſe of cold and red blood.

In the courſe of his reſearches, Cit. Cuvier has obſerved in the leech red blood, not that which it ſucks, and which would be contained in the inteſtinal canal, where it is immediately altered, but a real nouriſhing fluid contained in the veſſels, and circulating there by means of an alternate movement of the ſyſtole and the diaſtole. Theſe veſſels form four principal trunks, two of which are lateral, the third dorſal and the laſt ventral. The two former are of an order different from the two latter ; but the author has not yet been able to determine which are the arterial, and which the venal. The two lateral veſſels proceed from one end of the body to the other, and are joined by branches, which form a very beautiful tiſſue when injected. The ventral and dorſal veſſels do not form a tiſſue of the like kind. They only throw out branches diſpoſed alternately in an oblique direction, and ſubdivided in the uſual manner. The ſecond is placed exactly under this medullary cord of the ganglions, from which all the nerves proceed. It is impoſſible to open a leech, without producing a great effuſion of that blood. Enough however remains in the veſſels to be diſtinguiſhed. Its colour is almoſt the ſame as that of the arterial blood of the frog.

The whole of this original work is treated in a manner by no means derogatory to the fame of this author, who is already known to us as an excellent naturaliſt.

*Hiſtoire Naturelle des Poiſſons, &c.* i. e. " *The Natural Hiſtory of Fiſhes ;*" by Citizen LACEPEDE. 4to. and likewiſe 12mo. *Paris.* Plaſſan.

This is, perhaps, the beſt work upon the ſubject that has ever been publiſhed, and may be conſidered as a valuable continuation of *Buffon.* It is written in a manner equally pleaſing and inſtructive. The moſt intereſting ſection of the book is that, in which the ingenious author conſiders the diſtinctive character between fiſhes and other animals, namely their cold red blood, their having no lungs, and reſpiring through the gills. After having ably commented upon theſe particulars, he treats of the ſenſes of fishes, the most perfect of which is that of smell, then the sight, and next to it, hearing; touch and taste being the most imperfect of their sensitive faculties. On the whole, he ranks them towards the middle of the scale, for ascertaining the degree of sensibility in such beings as are provided with organs of sense.

Cit. Lacépède further examines the respective degrees of re-production, the swimming power, the mode of nourishment, sleeping, and the social qualities of these animals: he concludes this part of the work by giving an account of the prevalent diseases among fishes, their frequently prodigious size, their utility, &c. In describing that astonishing animal the *torpedo*, the author satisfactorily illustrates the extraordinary effects, which the person attempting to seize it experiences on the arms;—when treating of the *shark*, he furnishes us with an animating picture of that voracious monster.

Throughout this classical work, the author is highly instructive, particularly on subjects where he endeavours to diffuse scientific knowledge; correct in his definitions and details; accurate in classing objects; luminous in the explanation of causes producing the phenomena of nature; interesting in painting habits and peculiarities; and his language is truly sublime when he demonstrates the difference subsisting between the various species of fishes, as well as the characteristic distinction prevailing between them and other animals.

*Tableau des Systêmes de Botanique, &c.—A View of the Systems of Botany, general and particular; the Principles on which they are founded: together with an Explanation of the Sexual System of Linnæus. By* MOUTON FONTENILLE. 8vo. *Lyons.* Reyman.

The title-page of this work affords a sufficient idea of its contents. We do not recollect that we possess in the English language a book of a similar nature, and equal merit. The two 'Memoirs' which the author has subjoined to this work, are not the least interesting part of it: the first contains a detailed account of experiments on various methods of drying plants, and their preservation in herbals; in the second we meet with observations on the different kinds and species of vegetables, peculiar to the calcareous and granite mountains in the vicinity of Grenoble. The whole reflects honour on the industry and abilities of the author, and will be read with satisfaction by the botanist, as well as the naturalist.

*Les Plautes, &c.—The Plants, a Poem.* By RENE RICHARD CASTEL, Professor of Literature, &c. Second edition, enlarged, 18mo. with five plates, (3 livres bound). *Paris.* Déterville.

This production of the French Muse has been universally admired, ever since its first appearance. The picturesque disposition of the plan, combined with elegance and sweetness of versification, have rendered it a work of general approbation: insomuch that the National Institute have deemed it worthy of being announced as a work of superior merit, at one of the late solemn festivals of the French Republic. The author has not been insensible of these honourable tokens of public attention. In the present edition, he has made considerable improvements, and added many harmonious verses. In short, this specimen of didactic poetry may be justly ranked in the first class of such compositions; for, whether in point of originality and flights of genius, or in the beauty and arrangement of imagery, it is in every respect a fair rival of Dr. DARWIN'S '*Botanic Garden.*'

To the explanatory notes, the author has likewise made numerous additions, so that they afford a variety of useful information, while they are written with elegance and precifion. Laftly, the engravings are correct and well executed.

*Hiftoire des Plantes de l'Europe, &c.—The Hiftory of European Plants; or Elements of Practical Botany: being a fummary defcription of the indigenous Plants, as well as the moft ufeful exotics; according to the arrangement and principles of Linnæus, and accompanied with modern obfervations.* By J. E. GILLIBERT. 2 vols. 12mo. *Lyons.* Leroy.

This elementary treatife, particularly the firft volume, is chiefly intended for the inftruction of the tyro, in the firft principles of botany, and to introduce him to the knowledge of the moft common plants. The fecond volume contains more extenfive defcriptions, and critical difcuffions of foreign plants. The whole is interfperfed with interefting obfervations on the different plants, both indigenous and exotic. The author has likewife added to this ufeful little work a table, exhibiting the fynonimous botanical names of plants ufed by TOURNEFORT and LINNÆUS, together with a pretty complete dictionary of technical terms, peculiar to the fcience of botany. The plan he has adopted deferves every commendation, and ought to be imitated not only in teaching natural hiftory, but in other ftudies, particularly in geography and univerfal hiftory, as it is unqueftionably the eafieft, and moft natural, to promote and facilitate the acquifition of fcience. And this fimply confifts in leading his pupils, by gradual fteps, from the native plants growing wild in the vicinity of Lyons, to thofe of other and more diftant parts of France; from thefe, to the knowledge of the vegetable productions of the different countries of Europe, and laftly, to the plants peculiar to the moft diftant countries.

*Plantæ novæ, &c.—A Defcription of new plants which grow wild in Spain, or are cultivated in the gardens of that kingdom.* By D. A. CAVANILLES. Folio. Vol. I.. and II. *Madrid.*

The firft volume of this magnificent collection contains 110 plates, among which we meet with reprefentations of feveral new fpecies of plants; the fecond is embellifhed only with 34 plates, but they exhibit moftly new, rare, and valuable plants, natives either of Spain or Mexico. The defcriptions appear to be critically accurate, and relate to the ftalk, leaves, flowers, calyx, corolla, ftamina, buds, fruit, and feed of each plant; they particularize the places where it grows naturally, and by artificial culture, whether it is an annual or perennial plant; the time of its flowering; including many ufeful and interefting obfervations. Nothing, indeed, feems to be omitted, which could contribute to render this national work, the firft of its kind in Spain, a complete hiftory of the different plants therein enumerated.

*Plantes rares du Royaume de Naples.—The fcarce Plants of the Kingdom of Naples.* By DOMINIQUE CYRILLI; 2 Numb. Folio. *Naples.*

This is an uncommonly elegant and learned work. The language, although not ftrictly claffical, is generally correct; the defcriptions are clear and fatisfactory; and many of the plants defcribed are equally curious and interefting: among thefe we take particular notice of the *braffica fruticulofa*, which grows on the dry fea-fands near Naples; the *allium trifoliatum*, a fpecies hitherto unknown; the *allium fpeciofum*, and the *allium cilliatum*, of which two laft fpecies he gives the hiftory, together with their botanical characters.

The fecond number concludes with a plant, which the author terms *arundo ampelodefmon*; it is a fpecies of the calamus, frequently found on the coaft of Naples and Sicily; and Mr. Cyrilli affures us, that this vegetable promifes incalculable incalculable advantages to the agriculturist, the mariner, and the artist. Each number of this collection is accompanied with twelve well-engraved plates.

*Herbarium Mauritianum.*—"*The Mauritian Herbal*": *by* P. R. WILLEMET, with a Preface, by A. L. MILLIN. 8vo. *Leipzig.* Wolf.

The herbal before us is the fruit of a journey to India, undertaken by Cit. WILLEMET, with a view to explore and illustrate this part of natural history. It contains analytical descriptions of no less than 200 Mauritian plants, together with their botanical and vulgar names. The author points out the particular situations in the island of Mauritius, where these plants grow spontaneously; he mentions all the varieties he met with, and ascertains the time when they are in flower: a number of these are new, and first discovered by himself. The premature death of this ardent and able botanist, will be much and long regretted, particularly by the lovers of that useful and agreeable branch of the sciences.

